# Hybrid quantum neural network models for fruit quality assessment

**DOI:** 10.1371/journal.pone.0332528

**Published:** 2025-12-10

**Authors:** Danish ul Khairi, Kamran Ahsan, Syed Zeeshan Ali, Wadee Alhalabi, Somayah Albaradei, Muhammad Shahid Anwar

**Affiliations:** 1 Department of Computer Science, Federal Urdu University of Arts Science and Technology, Karachi, Pakistan; 2 Immersive Virtual Reality Research Group, Department of Computer Science, King Abdulaziz University, Jeddah, Saudi Arabia; 3 Department of Computer Science, Faculty of Computing and Information Technology, King Abdulaziz University, Jeddah, Saudi Arabia; 4 IRC for Finance and Digital Economy, King Fahd University of Petroleum and Minerals, Dhahran, Saudi Arabia; Commonwealth Scientific and Industrial Research Organisation, AUSTRALIA

## Abstract

This study investigates hybrid quantum neural networks for fruit quality assessment, with a focus on the impact of the entangling gate choice. Two architectures were developed: NNQEv1, utilizing controlled-NOT (CNOT) gates, and NNQEv2, employing controlled-phase (CZ) gates. A theoretical justification is provided, based on gate decomposition and hardware-aware noise considerations, suggesting the CZ-based architecture is likely to be more stable. The performance of the models was evaluated through the computational execution of their quantum circuits on classical hardware and compared against classical and state-of-the-art deep learning models. The proposed models demonstrated competitive performance, achieving test accuracies of 98.7% on MNIST, 98.6% on the FruitQ dataset, and 96.7% on a custom, data-scarce Apple dataset. The experimental results align with the theoretical analysis: the CZ-based NNQEv2 model, when compared to the CNOT-based NNQEv1, consistently showed more stable training dynamics and yielded tighter confidence intervals in cross-validation. This work presents a foundational, computational study on the role of gate-level design choices, intended to inform the development of future quantum machine learning algorithms.

## 1 Introduction

The rising demand for high-quality food products has made effective quality assurance increasingly important. While traditional inspections rely on labor-intensive human visual checks, a variety of automated, vision-based techniques have been introduced, including RGB, hyperspectral, and thermal imaging [1–5]. Among these, computer vision combined with machine learning has been successfully applied to fruit grading and quality assessment [6,7]. However, the growing complexity of agricultural data poses significant scalability and real-time processing challenges for classical deep learning models like Convolutional Neural Networks (CNNs) [8,9].

Quantum Machine Learning (QML) has emerged as a promising paradigm to address some of these fundamental challenges [10,11]. By leveraging principles of superposition and entanglement, QML algorithms possess the theoretical potential to explore high-dimensional feature spaces more efficiently than their classical counterparts [12,13]. A particularly viable path for near-term applications lies in Hybrid Quantum-Classical Neural Networks (HQNNs), which combine the feature-extraction power of parameterized quantum circuits (PQCs) with the robust learning frameworks of classical neural networks [14]. This approach is actively being explored across diverse domains, including regression tasks, satellite mission planning, and personalized medicine, demonstrating its broad potential [15–18].Despite these advancements, a notable gap remains in the literature. Most HQNN research focuses on high-level architectural innovation or proof-of-concept demonstrations [14,19], with limited attention to the hardware-aware design choices that could critically influence performance. A particularly underexplored aspect is the choice of two-qubit entangling gates, such as the Controlled-NOT (CNOT) and Controlled-Z (CZ) gates, which are fundamental to generating quantum correlations. Theoretical insights into fault-tolerant quantum computing indicate that these gates interact with noise differently—CNOT gates may propagate local dephasing errors into non-local correlated errors [20,21], while CZ gates, being diagonal, avoid this specific error propagation [22].

However, the majority of current research on HQNNs for image classification focuses on high-level architectural design or proof-of-concept demonstrations [19]. A critical, yet often overlooked, aspect is the impact of low-level, hardware-aware design choices on model performance. The choice of the two-qubit entangling gate—the fundamental building block for generating quantum correlations—is paramount, especially on Noisy Intermediate-Scale Quantum (NISQ) devices. While gates like Controlled-NOT (CNOT) and Controlled-Z (CZ) are logically interconvertible, their physical implementations and resilience to noise can differ dramatically. The non-commuting nature of the CNOT gate with dephasing noise, a dominant error source, can lead to the propagation and transformation of errors, a phenomenon that can degrade algorithmic performance [20,21]. In contrast, the CZ gate’s diagonal structure provides inherent robustness against this specific noise channel [20,22]. This observation aligns with developments in other QML applications, where hardware-aware gate selection has proven crucial for achieving reliable results [23].

This gap in understanding—the direct link between entangler choice and noise resilience—motivates the present work. This study systematically investigates the impact of CNOT versus CZ entanglement within a hybrid quantum-classical framework for fruit quality assessment. A rigorous theoretical justification is provided to hypothesize why the CZ-based architecture should exhibit more stable and generalizable performance. Two models, NNQEv1 (CNOT-based) and NNQEv2 (CZ-based), are introduced and their performance is evaluated in ideal, noiseless simulations on benchmark and custom datasets. To contextualize their performance, the models are benchmarked against both classical machine learning methods and state-of-the-art deep learning architectures.

The primary contributions of this work are:

- A rigorous theoretical analysis justifying why the CZ gate is expected to yield more robust performance in noisy environments, based on native gate decomposition and noise propagation models.
- A systematic comparison of CNOT- and CZ-based models via quantum simulation on classical hardware, demonstrating a measurable impact of the entangler choice on model generalization and stability.
- A comprehensive benchmarking of the proposed HQNNs against both classical baselines and state-of-the-art deep learning models to contextualize their performance.
- An evaluation on a data-scarce custom Apple dataset to explore the utility of HQNNs in specialized domains where data limitations can hinder the performance of classical models.

Through this focused investigation, this paper moves beyond a general exploration of HQNNs to provide specific, hardware-aware insights crucial for designing the next generation of effective and robust QML algorithms. This work aims to confirm that the choice of entangling gate is a critical factor in model performance, pointing to a future where carefully designed hybrid quantum methods can help overcome computational challenges in agritech.

The structure of the paper is as follows: Sect 1 is an introduction, and Sect 2 is a literature review and related works. Sect 3 describes the materials and methods, including the proposed model. Sect 4 details the experimental results, encompassing datasets, data preprocessing, model performance comparisons, and training/testing procedures. Sect 5 provides the conclusion and highlights the future direction.

## 2 Related works

The automated classification of fruit quality has evolved significantly, transitioning from traditional machine learning approaches to deep learning and, more recently, quantum-enhanced methodologies. Early studies predominantly employed algorithms such as Support Vector Machines (SVMs), k-Nearest Neighbors (KNN), and Decision Trees (DT) [24], which relied on manually engineered features—color, texture, and shape—extracted from different color spaces, including RGB, HSV, and L*a*b. While these methods achieved reasonable results, their performance was inherently constrained by the quality and representativeness of the hand-crafted features.

The advent of deep learning, particularly Convolutional Neural Networks (CNNs), brought a paradigm shift. Architectures like Inception-V3, ResNet, and Vision Transformers (ViT) demonstrated the ability to learn hierarchical feature representations directly from pixel data [25–28], effectively eliminating the need for manual feature extraction. This enabled state-of-the-art accuracies in fruit quality assessment, as seen in Table 1. However, these models often require large datasets and substantial computational resources, limiting their scalability and applicability in domains with scarce data [9].

**Table 1:** Apple quality classification using different methodology systems.

Ref	Color Space	Processing Methodology	Accuracy (%)	QML
[25]	RGB	Inception-V3	91.2	X
[44]	RGB+HSI	OFPs	95	X
[26]	RGB	OB-Net	95.64	X
[27]	RGB	IMP-ResNet50	96.5	X
[25]	GrayScale	GLCM/HOG+SVM	98.98	X
[28]	RGB	ViT CNN	99.5	X
[24]	RGB, HSV, L a b	ANN, DT, SVM, KNN	89.8, 90.8, 93, 90	X
Ours	Greyscale	Quantum Circuit (QC) + NN	96.67	✓

Quantum Machine Learning (QML) has recently emerged as a potential solution to some of these limitations. Leveraging quantum phenomena, QML models can, in principle, access exponentially large feature spaces and model complex correlations that classical approaches may fail to capture [12,13]. In particular, Hybrid Quantum-Classical Neural Networks (HQNNs) integrate Parameterized Quantum Circuits (PQCs), or Variational Quantum Circuits (VQCs), with classical neural networks for optimization and classification [29,30]. Initial applications, such as Quantum Convolutional Neural Networks (QCNNs), have shown promise in image classification tasks. Despite this promise, a critical area remains underexplored: the impact of low-level, hardware-aware gate choices. As summarized in Table 2, recent literature highlights that while CNOT and CZ gates are logically similar, their physical implementations yield significant differences in fidelity and noise resilience across various hardware platforms [31–37]. However, the direct consequence of this gate-level choice on the end-to-end performance of a hybrid image classification model has not been systematically studied. This represents a critical research gap, as the practical success of any NISQ-era algorithm will depend heavily on such noise-aware design decisions. Table 2 summarizes comparative findings from recent literature, underscoring the higher fidelity and noise resilience of CZ gates in various hardware platforms.

**Table 2:** Comparison of CNOT and CZ gate fidelity and noise characteristics.

Paper Title	Year	Key Findings on CNOT and CZ Gate Fidelity and Noise
High-fidelity parallel entangling gates on a neutral-atom quantum computer [39]	2023	Achieves 99.5% fidelity for CZ gates on neutral atoms, operating on up to 60 qubits; CZ outperforms typical superconducting CNOT/CZ fidelities due to better noise resilience in parallel operations.
Quantum process tomography of two-qubit controlled-Z and controlled-NOT gates using superconducting phase qubits [40]	2010	CZ gates simpler to implement with higher fidelity than CNOT; less susceptible to noise, making CZ preferable for robust entangling in superconducting qubits.
Fast high-fidelity entangling gates for spin qubits in Si double quantum dots [41]	2019	CNOT and CZ-like (CPHASE) gates achieve >99.99% fidelity with 45 ns gate times; CZ benefits from simpler control pulses, reducing noise in silicon spin qubits.
Towards a Quantum Hardware Roofline: Evaluating the Impact of Gate Expressivity on Quantum Processor Performance [42]	2024	CNOT fidelity at 0.9968, but CZ shows similar expressivity with lower error accumulation due to fewer gates; error rates $\sim 10^{-2}$ affected by crosstalk and decoherence.
Leveraging biased noise for more efficient quantum error correction on neutral-atom Rydberg platforms [43]	2025	Bias-preserving CZ gates reduce logical error rates in surface codes compared to CNOT, leveraging biased noise for better error correction.
Noise-Tolerant Superconducting Gates with High Fidelity [6]	2023	Reports 94.6% fidelity for CNOT and 97.8% for CZ via quantum process tomography; CZ exhibits higher fidelity and noise tolerance.
Depth Optimization of CZ, CNOT, and Clifford Circuits [1]	2022	Shallower CZ circuits reduce noise accumulation, improving fidelity over CNOT in NISQ hardware by minimizing decoherence exposure.

To contextualize this research gap, recent advances in QML for data-scarce domains are considered. The work by Wang et al. [38] demonstrated the effective application of Quantum Kernel methods to a regression task using structured, tabular data from semiconductor fabrication. The present study, however, explores a complementary path by addressing an image classification task. A Variational Quantum Circuit (VQC) is employed as a trainable feature extractor for image patches, with the resulting features processed by a classical neural network. The novelty of this study lies not in a high-level architectural innovation, but in a foundational, hardware-aware investigation. A rigorous theoretical and empirical analysis is provided on how the low-level choice of entangling gate (CNOT vs. CZ) fundamentally impacts the stability and generalization of the extracted visual features. This gate-level design focus for image-based classifiers represents a critical and distinct contribution, providing essential insights for building more robust and reliable VQC-based models for near-term quantum hardware.

Our work addresses this research gap by presenting a systematic comparative analysis of CNOT and CZ gates within HQNNs for a real-world fruit classification task. This approach not only assesses classification performance but also examines the influence of gate choice on model robustness, thereby contributing new insights into practical QML system design.

## 3 Materials and methods

**Fig 1:**
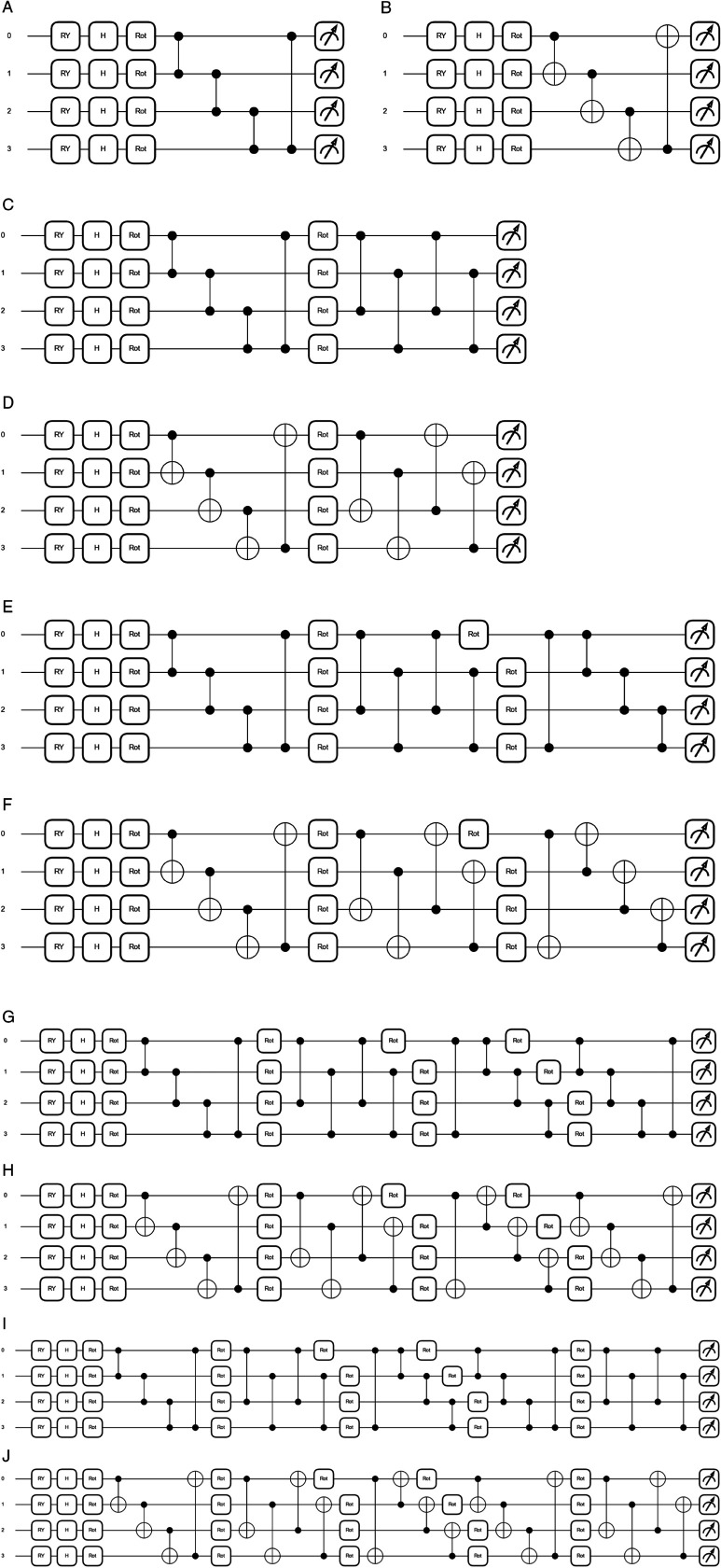
Quantum circuit diagrams for different layers and entangling gates: (a) Layer 1 with NNQEv2 entangler. (b) Layer 1 with NNQEv1 entangler. (c) Layer 2 with NNQEv2 entangler. (d) Layer 2 with NNQEv1 entangler. (e) Layer 3 with NNQEv2 entangler. (f) Layer 3 with NNQEv1 entangler. (g) Layer 4 with NNQEv2 entangler. (h) Layer 4 with NNQEv1 entangler. (i) Layer 5 with NNQEv2 entangler. (j) Layer 5 with NNQEv1 entangler.

This research investigates the application of quantum neural networks (QNNs) for image classification, employing a hybrid quantum-classical architecture to leverage the unique capabilities of both quantum and classical computing paradigms. Two distinct QNN architectures, NNQEv1 and NNQEv2, are designed and compared to assess the impact of different entanglement strategies on classification performance. Both architectures utilize a quantum circuit for feature extraction, followed by a classical convolutional neural network (CNN) for final image classification. The quantum circuit processes 2×2 pixel patches extracted from the input images, transforming these classical pixel values into quantum representations. As depicted in Fig 1, both NNQEv1 and NNQEv2 follow a common circuit block structure shown in Fig 1, differing only in the entanglement gates used.

The initial step within the quantum circuit is the **encoding layer**, where each pixel value *x*, constrained within the range 0≤x≤1, is mapped to a quantum state through the application of an *RY* rotation gate. Specifically, the pixel value is encoded into the rotation angle θ=πx, yielding a quantum state $|\psi_{j}\rangle =RY(\pi x_{j})|0\rangle$, where *x*_*j*_ represents the pixel value corresponding to qubit *j*. The mathematical representation of the *RY* gate is defined in Eq (1).

Following the encoding, a superposition layer is implemented by applying a Hadamard gate (*H*) to each qubit, creating an equal superposition of |0⟩ and |1⟩ states, represented as $|\psi_{j}\rangle =H|\psi_{j}\rangle$. The Hadamard gate is represented in Eq (2).

Subsequently, Entanglement layers, ranging from one to five, are introduced to establish quantum correlations. This study deliberately employs CNOT and CZ gates for comparative analysis due to their fundamental roles and distinct characteristics in quantum computing. CNOT gates, being universal and parameter-efficient, serve as a baseline for entanglement, facilitating a broad understanding of its effects within QNNs. In contrast, CZ gates, known for high-fidelity operations [45], enable an analysis of enhanced entanglement quality and its impact on classification performance. Their robustness against errors in NISQ devices further supports this investigation. CNOT and CZ represent state-dependent and phase-dependent entanglement, respectively, allowing for a focused evaluation of their influence on QNN performance. NNQEv1 employs CNOT gates, while NNQEv2 utilizes CZ gates, as defined in Eqs (3) and (4). This binary comparison isolates entanglement effects, providing insights into efficient quantum-enhanced image classification models [45].The choice of CNOT versus CZ gates is motivated by their distinct properties and behavior on realistic hardware. For instance, on many superconducting platforms that use a cross-resonance interaction, transpilers can synthesize CZ gates more efficiently with fewer native gates, which can reduce decoherence. Following the entanglement layers, **parameterized rotation layers**, implemented using Strongly Entangling Layers, apply three-axis rotations (R(θ,ϕ,λ)=RZ(λ)RY(ϕ)RZ(θ)) to each qubit. The *RZ* and *RX* gates, components of the three-axis rotation, are defined in Eqs (5) and (6). These layers introduce trainable parameters for fine-tuning the quantum feature extraction process. The quantum circuit with up to five layers (*n* = 5) is designed to enhance image processing capabilities as shown in Fig 1. Finally, the measurement layer collapses the quantum states into classical values by measuring the expectation value of the Pauli Z operator (*Z*), yielding $\langle Z_{j}\rangle =\langle \psi_{j}|Z|\psi_{j}\rangle$. The Pauli Z gate is defined in Eq (7). This transforms the quantum features into a format suitable for the classical CNN.

The classical CNN, which performs the final image classification, comprises convolutional layers with ReLU activation, MaxPooling layers, a flatten layer, and dense layers with softmax activation. The observation that increasing the number of quantum entanglement layers did not significantly improve image classification accuracy is noted and will be further discussed.

### 3.1 Mathematical formulation of the quantum feature extractor

To provide a formal understanding of the quantum feature extraction process, this section presents a mathematical description of the circuit structure. The formulation captures how grayscale image patches are encoded into quantum states, transformed through variational layers, and measured to extract classical features.

#### 3.1.1 Data encoding and state preparation.

The process begins with a classical input image $I\in {\mathbb{R}}^{H\times W}$. This image is divided into non-overlapping 2×2 patches, *P*_*j*,*k*_. Each patch is flattened into a 4-element vector of normalized pixel intensities:


$$\phi =(\phi_{0},\phi_{1},\phi_{2},\phi_{3})$$

where, for a patch with its top-left corner at position (*j*,*k*):


$$\phi_{0}=I_{j,k},\;\phi_{1}=I_{j,k+1},\;\phi_{2}=I_{j+1,k},\;\phi_{3}=I_{j+1,k+1}$$

The system is initialized in the 4-qubit ground state:


$$|0\rangle^{⊗4}$$

The classical pixel values are then encoded into the quantum state using a unitary operation, $U_{\text{enc}}$, which applies an *R*_*Y*_ rotation followed by a Hadamard gate to each qubit:


$$U_{\text{enc}}(\phi )=\overset{3}{\underset{i=0}{⨂}}H_{i}R_{Y}(\pi \phi_{i}{)}_{i}$$

$$|\psi_{\text{enc}}\rangle =U_{\text{enc}}(\phi )|0\rangle^{⊗4}$$

#### 3.1.2 Variational entangling layers.

The encoded state is then processed by $L=n_{\text{layers}}$ variational layers. Each layer consists of a single-qubit rotation unitary and a two-qubit entangling unitary. The single-qubit rotations are parameterized by a set of trainable angles, $\theta_{l,i,k}$, allowing the model to learn optimal feature transformations. The entangling unitary uses either CNOT (for NNQEv1) or CZ (for NNQEv2) gates. The structure of a single layer is given by:


$$U_{\text{layer},l}=U_{\text{entangler},l}\cdot \overset{3}{\underset{i=0}{⨂}}R_{Z}(\theta_{l,i,2}{)}_{i}R_{Y}(\theta_{l,i,1}{)}_{i}R_{X}(\theta_{l,i,0}{)}_{i}$$

The entanglement pattern, which defines the pairs of qubits to be entangled in each layer, follows a specific schedule designed to ensure comprehensive interaction between qubits across the layers:


$$U_{\text{entangler},l}=\left\{ \begin{array}{cc} \text{CNOT/CZ on }\{(0,1),(1,2),(2,3),(3,0)\} & l\in \{1,4\}\\ \text{CNOT/CZ on }\{(0,2),(1,3),(2,0),(3,1)\} & l\in \{2,5\}\\ \text{CNOT/CZ on }\{(0,1),(2,3),(1,2),(3,0)\} & l=3 \end{array}\right.$$

The full variational transformation is the product of these layers, forming the entangling unitary $U_{\text{entangle}}$:


$$U_{\text{entangle}}=\prod_{l=1}^{n_{\text{layers}}}U_{\text{layer},l}$$

This unitary is applied to the encoded state to produce the final state before measurement:


$$|\psi_{\text{final}}\rangle =U_{\text{entangle}}|\psi_{\text{enc}}\rangle$$

#### 3.1.3 Measurement and feature extraction.

To obtain classical features, the expectation value of the Pauli-Z operator ($\sigma_{z}$) is computed for each of the four qubits:


$$\langle \sigma_{z,i}\rangle =\langle \psi_{\text{final}}|\sigma_{z,i}|\psi_{\text{final}}\rangle ,\;i=0,1,2,3$$

These four expectation values form the feature vector for the corresponding input patch:


$$f_{j/2,k/2}=[\begin{array}{c} \langle \sigma_{z,0}\rangle \\ \langle \sigma_{z,1}\rangle \\ \langle \sigma_{z,2}\rangle \\ \langle \sigma_{z,3}\rangle \end{array}]\in {\mathbb{R}}^{4}$$

This process is repeated for all patches, creating a final output feature map *O* of size H/2×W/2×4, where each of the four channels corresponds to the expectation value of one qubit. This feature map is then passed to the classical CNN for final classification.


$$O_{j/2,k/2,c}=f_{j/2,k/2}[c],\;c=0,1,2,3$$

$$O\in {\mathbb{R}}^{H/2\times W/2\times 4}$$

#### Gate definitions.

The unitary matrices for the gates used throughout this work are defined as follows:

$$R_{Y}(\theta )=[\begin{array}{cc} \cos(\theta /2) & -\sin(\theta /2)\\ \sin(\theta /2) & \cos(\theta /2) \end{array}]$$ (1)

$$H=\frac{1}{\sqrt{2}}[\begin{array}{cc} 1 & 1\\ 1 & -1 \end{array}]$$ (2)

$$\text{CNOT}=[\begin{array}{cccc} 1 & 0 & 0 & 0\\ 0 & 1 & 0 & 0\\ 0 & 0 & 0 & 1\\ 0 & 0 & 1 & 0 \end{array}]$$ (3)

$$\text{CZ}=[\begin{array}{cccc} 1 & 0 & 0 & 0\\ 0 & 1 & 0 & 0\\ 0 & 0 & 1 & 0\\ 0 & 0 & 0 & -1 \end{array}]$$ (4)

$$R_{Z}(\lambda )=[\begin{array}{cc} e^{-i\lambda /2} & 0\\ 0 & e^{i\lambda /2} \end{array}]$$ (5)

$$R_{X}(\phi )=[\begin{array}{cc} \cos(\phi /2) & -i\sin(\phi /2)\\ -i\sin(\phi /2) & \cos(\phi /2) \end{array}]$$ (6)

$$\sigma_{z}=Z=[\begin{array}{cc} 1 & 0\\ 0 & -1 \end{array}]$$ (7)

#### Symbol definitions.

Key symbols used in the formulation are defined below for clarity.



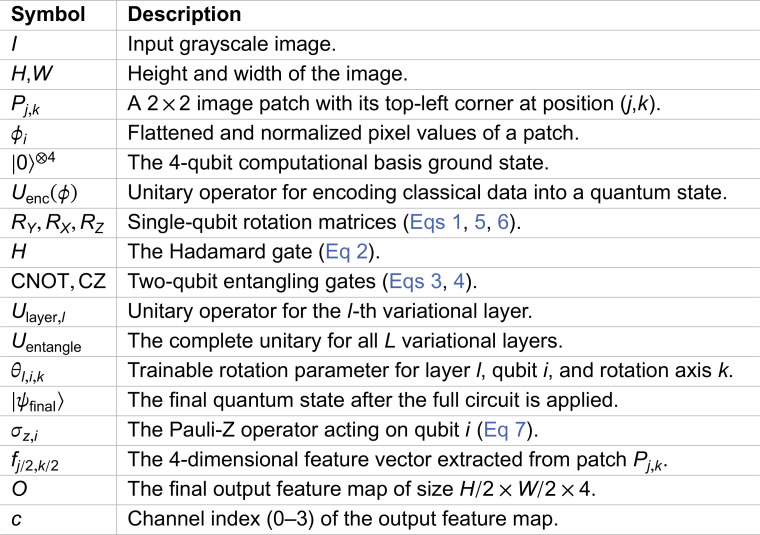



### 3.2 Theoretical analysis of entanglement strategies

A key objective of this study is to move beyond high-level architectural comparisons and investigate the impact of low-level, hardware-aware design choices. The selection of the two-qubit entangling gate is a fundamental decision that can critically influence a model’s performance, particularly in the context of noisy, intermediate-scale quantum (NISQ) hardware. This section provides a two-fold theoretical analysis of the CNOT and CZ gates, establishing a clear hypothesis for their expected performance differences.

#### 3.2.1 Efficiency of gate decomposition.

The performance of any algorithm on a NISQ device is highly dependent on the total number and depth of native hardware gates required for its implementation. Each gate introduces a source of error, and deeper circuits with more gates accumulate more noise, degrading performance. On many superconducting platforms, including IBM devices which use a cross-resonance interaction, the CNOT and CZ gates are not native operations and must be decomposed into a basis set of single-qubit rotations and a native two-qubit gate, such as the echoed cross-resonance (ECR) gate.

**Fig 2:**
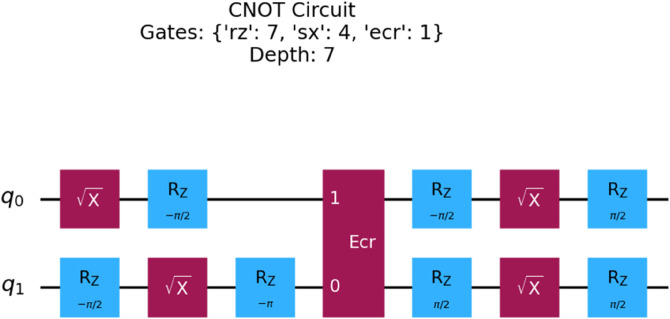
Transpiled CNOT quantum gate circuit showing the decomposition into 5 native gates with a circuit depth of 7.

**Fig 3:**
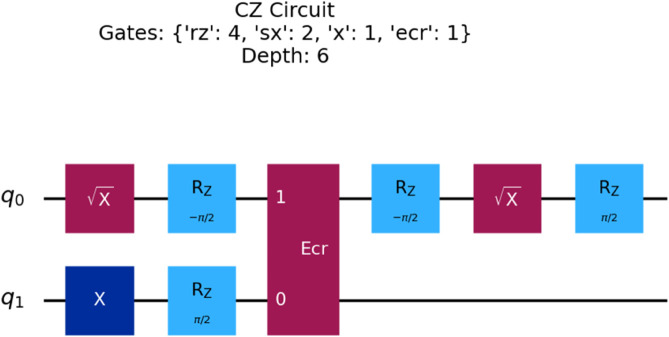
Transpiled CZ quantum gate circuit showing the more efficient decomposition into 3 native gates with a circuit depth of 6.

To obtain realistic hardware-aware metrics, the gate decompositions were analyzed using Qiskit’s transpiler with the properties of a specific hardware backend. For this purpose, the FakeBrisbaneV2 backend was used, which simulates the coupling map, basis gates, and other constraints of the real *ibm_brisbane* processor without running a full noise simulation. The CNOT gate is synthesized from native operations as:


CNOT=(I⊗(SX·RZ(−π/2)))·ECR·((RZ(π/2)·SX)⊗I),

which requires a sequence of five hardware-native gates (1 ECR, 2 SX, 2 RZ). Conversely, the CZ gate can be constructed more efficiently:


CZ=(I⊗RZ(π))·ECR·(RZ(−π/2)⊗I),

using only three hardware-native gates (1 ECR, 2 RZ). These decompositions, visualized in Figs 2 and 3, confirm that the CZ-based transpiled circuits are both shallower and involve fewer single-qubit gates. This 40% reduction in native gates per entangling operation provides a primary, structural advantage for the CZ-based NNQEv2 model, as it is expected to have a lower baseline error rate from accumulated gate errors.

#### 3.2.2 Inherent resilience to dephasing noise.

Beyond gate count, a more fundamental advantage of the CZ gate lies in its interaction with dephasing noise, a dominant and particularly damaging error channel in NISQ-era devices. Dephasing errors, which corrupt the relative phase of a qubit’s superposition without causing bit-flips, are modeled by the Pauli-Z operator. The way an entangling gate propagates this common error is critical to its robustness.

The error probability for any gate is related to the device’s coherence times, typically given by:


$$P_{err}\approx 1-e^{-t/T_{1}}\;(\text{amplitude damping})\;\text{or}\;1-e^{-t/T_{2}}\;(\text{dephasing}),$$

where *t* is the gate duration and $T_{1},T_{2}$ are the relaxation and dephasing times, respectively. The interaction with dephasing (T2) errors is particularly relevant here.

For the CZ gate, which is a diagonal matrix, a Z-error on the target qubit commutes with the gate operation:


CZ·(I⊗Z)=(I⊗Z)·CZ

This commutation is significant because it means a dephasing error on one qubit remains a localized, single-qubit error. It does not propagate to the control qubit, thus preserving what is known as error locality [20]. This property is crucial for mitigating the impact of noise.

In stark contrast, the CNOT gate transforms and spreads this same error across both qubits:


CNOT·(I⊗Z)=(Z⊗Z)·CNOT

In this case, a single-qubit dephasing error on the target is transformed into a **two-qubit correlated ‘ZZ‘ error**. This error-spreading mechanism amplifies the impact of noise, turning a simple, local error into a more complex, non-local one that affects a larger part of the quantum state and is more difficult to mitigate [20].

#### 3.2.3 Central hypothesis.

Based on this theoretical analysis of both gate decomposition efficiency and noise propagation dynamics, a clear, testable hypothesis is formulated: The architectural advantages of the CZ gate should manifest as measurably enhanced stability and generalization. The remainder of this paper is dedicated to empirically testing this hypothesis through a comprehensive computational study performed on classical hardware.

### 3.3 Data preprocessing and training methodology

The datasets were preprocessed through normalization of pixel values to the range [0,1], resizing, cropping, grayscale conversion, and extraction of non-overlapping 2×2 patches for quantum encoding. Data augmentation techniques were applied exclusively to the classical machine learning (ML) and deep convolutional neural network (DCNN) models to enhance their training diversity. In contrast, the quantum models did not employ explicit augmentation, as the quantum encoding process inherently generates four distinct output images per single input image, effectively augmenting the data within the quantum feature space. This approach ensures consistent evaluation of the pure impact of quantum encoding and entanglement strategies without confounding effects from external augmentation.

The training procedure simultaneously optimizes the parameters of the quantum circuits and the weights of the classical CNN components using supervised gradient-based learning methods. To assess the influence of various optimization algorithms on model performance, four distinct optimizers—Adam, RMSprop, SGD, and Adamax were employed during training. The comparative results, including accuracy and loss metrics, are summarized in Table 4, facilitating identification of the most effective optimizer for the hybrid quantum-classical architecture.

A 5-fold cross-validation strategy was adopted to ensure robustness and reliability in model evaluation, as well as to mitigate risks of overfitting and underfitting. The dataset was partitioned into five subsets, with each subset serving once as the validation set while the remaining four subsets were used for training. The average accuracy across all five folds is reported to provide a comprehensive estimate of model generalization capability.

## 4 Experimental results

### 4.1 Experimental setup

All simulations were conducted on a local workstation (Acer Predator Helios 300) equipped with an Intel Core i7 processor, 16GB RAM, and an Nvidia GTX 1080 GPU running Windows 10. The research was implemented in Python 3.8. The hybrid quantum-classical models were constructed and trained using the PennyLane, which integrated with Qiskit as the backend for quantum circuit simulation. For the theoretical analysis of hardware-specific gate decompositions in Sect 3.2.1, Qiskit’s transpiler was configured with the FakeBrisbaneV2 backend to simulate realistic architectural constraints. The classical components of the hybrid models, as well as the training of all baseline and State-of-the-Art (SOTA) machine learning models, were implemented and managed using TensorFlow. The four different optimizer are used Adam, SGD, RMSporp and Adamax with a learning rate of 0.001 over 40 epochs, based on the performance comparison in Table 4.

### 4.2 Datasets

The study used two benchmark datasets, MNIST (Modified National Institute of Standards and Technology) and FruitQ, for model evaluation and fruit quality assessment. A self-created Apple dataset was also tested for Apple quality classification using NNQE models. The training and test sets are dispersed at 80% and 20%, respectively.

#### 4.2.1 MNIST Digit dataset preparation.

**Fig 4:**
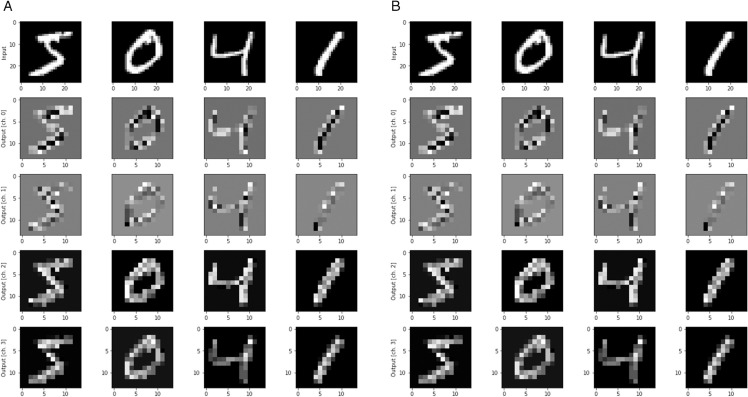
(a) CNOT Encoded Features (b) CZ Encoded Features.

MNIST, a database of handwritten digits (0 to 9), features 28x28 pixel images for neural network classification. Widely employed for machine learning initiation, it serves as a benchmark for testing neural network models, including those with Quantum machine learning models (HQNN, VQCs). The dataset contains 70,000 images, with 60,000 for training and 10,000 for testing. In some cases, reducing images may expedite training. Fig 4 visually depicts the dataset and features extracted using NNQEv1 and NNQEv2 circuits.

#### 4.2.2 FruitQ dataset.

**Fig 5:**
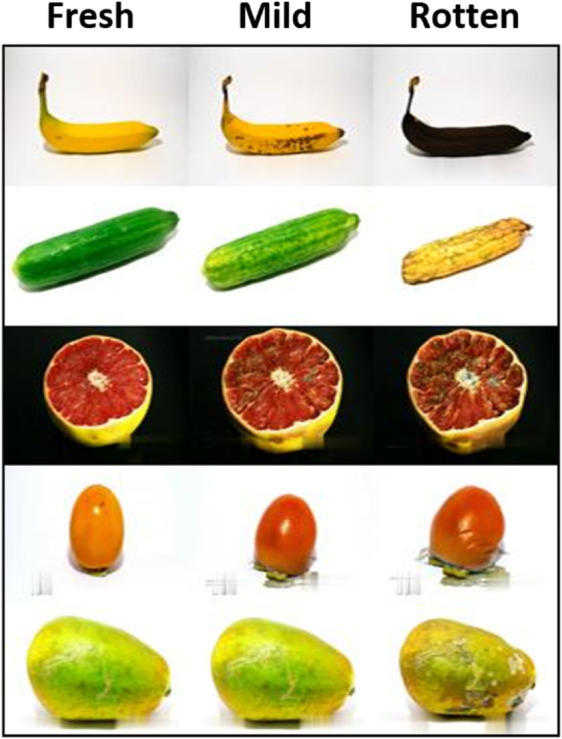
Sample images of FruitQ dataset.

The FruitQ dataset is used to classify fruit freshness levels into three categories: good, mild, and rotten. The dataset was collected using image frame extraction from the YouTube videos. The dataset contains eleven(11) varieties of fruits and is annotated according to the quality of fruit freshness as fresh, mild, and rotten. A total of 9421 images were collected, which are further divided into 3010 images for fresh, 2376 images for mild, and 4035 images for rotten class, as shown in Fig 5. The dataset is resized to 100 x 100 image pixels, then converted to a grayscale image and normalized the pixel value before feeding to the quantum convolution. The dataset is created specifically to monitor the freshness evaluation of fruits by performing machine learning methods in classification and detection tasks.

#### 4.2.3 Custom dataset and dataset preparation.

**Fig 6:**
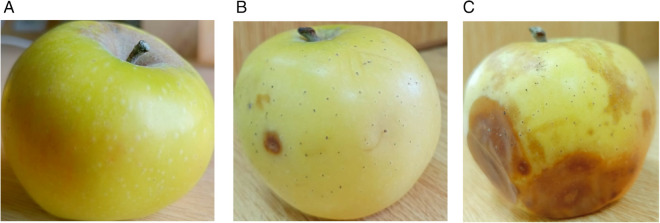
(a) Fresh Apple (b) Mid rotten Apple (c) Rotten Apple.

A custom dataset of apple images was developed to test the proposed models on a specific agritech-related task. The dataset supports classification across three distinct quality stages: fresh, mid-quality, and rotten, with visual examples provided in Fig 6. The images were captured in various local markets across Pakistan using a 13-megapixel smartphone camera (Infinix HOT 9 Play), ensuring diverse lighting conditions and angles for the collected samples.

The dataset comprises 150 carefully curated images, with a balanced representation of 50 images per class. During preprocessing, each image was manually annotated, cropped to isolate the apple, resized to a standardized 100 × 100-pixel resolution, and converted to grayscale. The pixel values were then normalized to the range [0, 1].

Given the small size of this dataset, which poses a significant risk of overfitting when training deep learning models, data augmentation was employed to enhance model generalization. This is a standard and necessary procedure for improving robustness on limited data. The following augmentation parameters were applied:

- Rotation Range: 15 degrees
- Width and Height Shift Range: 0.1
- Shear Range: 0.1
- Zoom Range: 0.1
- Horizontal Flip: True
- Fill Mode: ’nearest’

It is explicitly acknowledged that this dataset is small; its purpose is to serve as a challenging **test case for a data-scarce domain**. The use of both data augmentation and 5-fold cross-validation are methodological choices made specifically to ensure a robust and reliable performance evaluation despite the limited number of unique samples.

A visual sample of the dataset is provided in Fig 6, illustrating the variation in apple conditions. This structured approach ensures consistency while allowing for reliable performance assessment in classification tasks.

**Fig 7:**
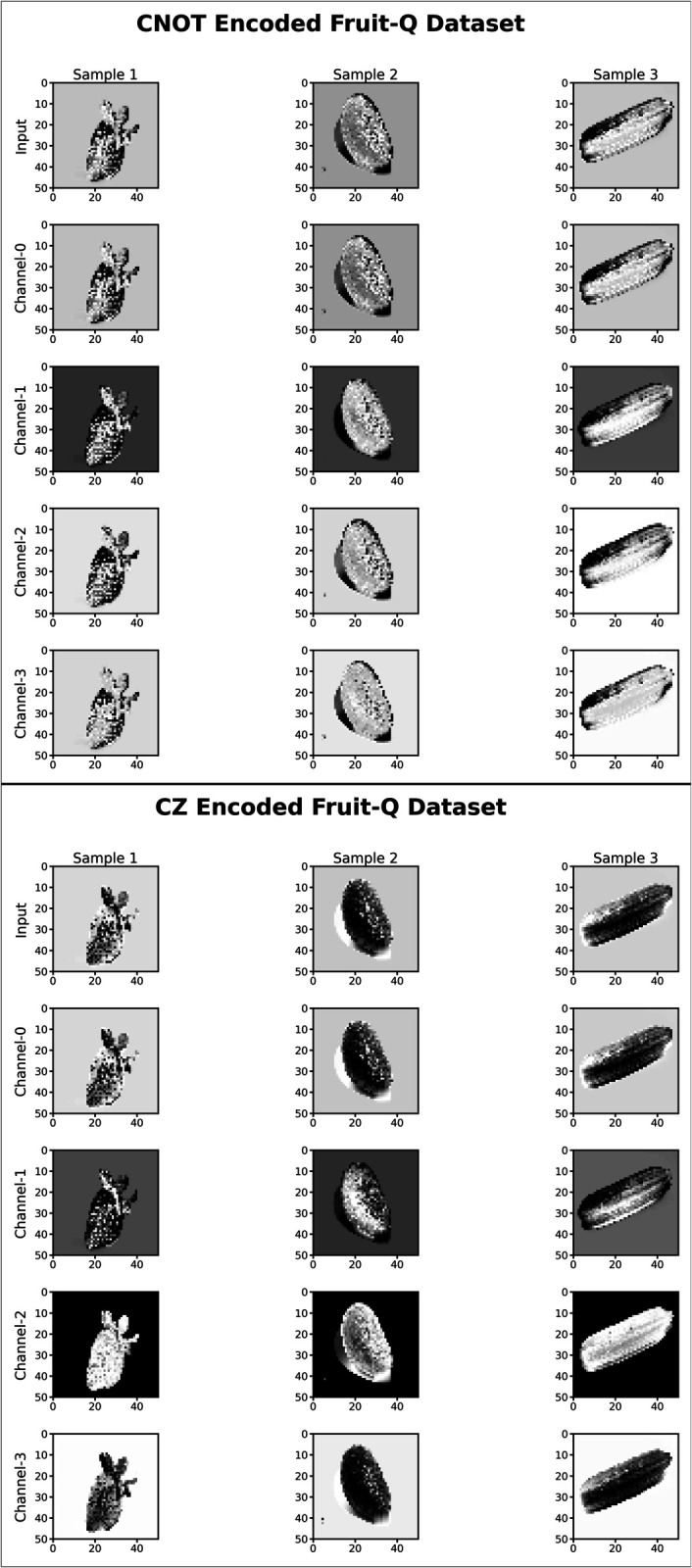
CNOT encoded features and CZ encoded features.

**Fig 8:**
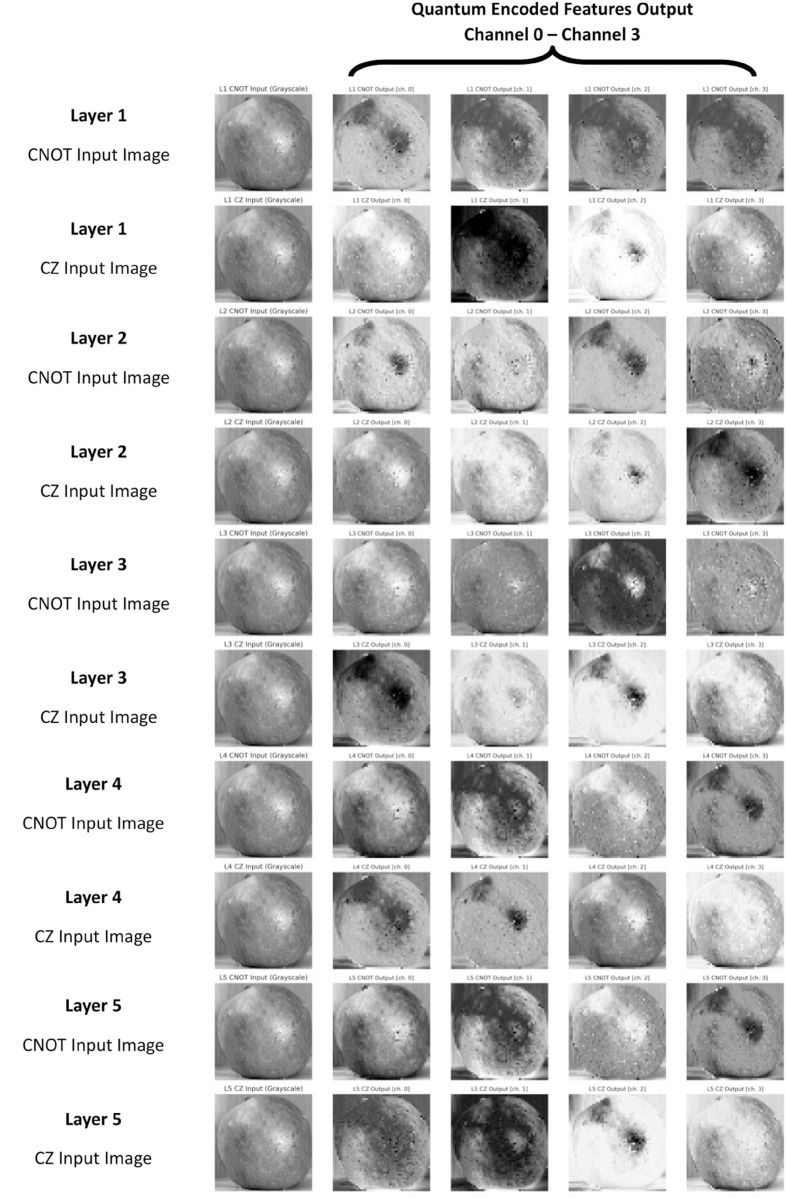
Visual comparison of quantum-encoded feature maps generated by the NNQEv1 (CNOT) and NNQEv2 (CZ) entanglers for a single input image. The subtle differences in texture and feature highlighting emerge from the different entanglement strategies.

Figs 7 and 8 present a comparative analysis of feature outputs obtained using the NNQEv1 and NNQEv2 circuits for quantum image processing. The Fig 8 illustrates five distinct quantum circuit configurations (Layers 1 to 5), each layer encoded using CNOT or CZ entanglers. Each configuration generates four output images corresponding to different measurement channels. This visualization provides insights into how these quantum gates transform image data within the quantum computing framework.

### 4.3 Model performance evaluation

**Fig 9:**
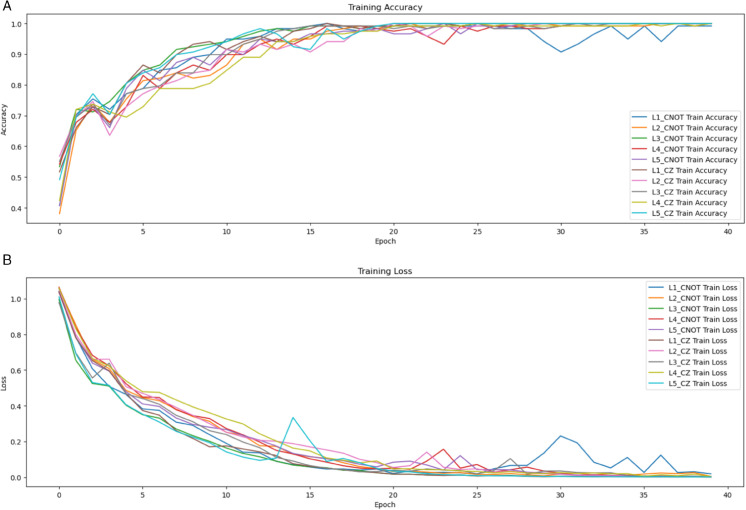
Training accuracy and loss for NNQEv1 and NNQEv2 across 5 layers and 40 epochs.

The Neural Network Quantum Entanglement architectures NNQEv1 and NNQEv2 underwent a comprehensive simulation-based evaluation on the three datasets. To provide a rigorous performance context, these hybrid models were benchmarked against a suite of classical and quantum algorithms. This included standard machine learning baselines (Support Vector Machines, k-Nearest Neighbors), a purely quantum SVM variant, and, crucially, two state-of-the-art classical deep learning models: EfficientNetB0 and VGG16, whose results are included for the FruitQ and Apple datasets. This comprehensive benchmarking allows for a clear assessment of the hybrid models’ competitiveness in various scenarios. All performance metrics, derived from 5-fold cross-validation, are detailed in Table 3, while Fig 9 illustrates the training dynamics

**Table 3:** Table comprehensive performance comparison of classical, quantum, and hybrid models across datasets.

Dataset	Model	Train Acc.	Test Acc.	Precision	Recall	F1 Score	Mean CV Acc.	Confidence Interval (CI)
**MNIST Digit**	NNQE_v1 (CNOT)	0.9986	0.9858	0.9859	0.9858	0.9858	0.9813 ± 0.0015	(0.980, 0.983)
	NNQE_v2 (CZ)	0.9993	0.9872	0.9873	0.9872	0.9872	0.9816 ± 0.0005	(0.981, 0.982)
	Quantum SVM	–	–	–	–	–	–	–
	SVM (linear)	1.0000	0.9778	0.9779	0.9778	0.9777	0.9708 ± 0.0105	(0.960, 0.981)
	SVM (poly)	0.9979	0.9917	0.9917	0.9917	0.9917	0.9882 ± 0.005	(0.983, 0.993)
	SVM (rbf)	0.9965	0.9861	0.9862	0.9861	0.9861	0.9861 ± 0.008	(0.978, 0.994)
	SVM (sigmoid)	0.9068	0.9000	0.9023	0.9000	0.9006	0.9033 ± 0.0245	(0.879, 0.928)
	KNN (k=3)	0.9930	0.9833	0.9835	0.9833	0.9832	0.9847 ± 0.0125	(0.972, 0.997)
	EfficientNetB0 [46]	-	0.981	-	-	-	-	(-, -)
	VGG16 [47]	-	0.9735	-	-	-	-	(-, -)
**Fruit Q**	NNQE_v1 (CNOT)	0.9933	0.9867	0.9888	0.9867	0.9867	0.9866 ± 0.0143	(0.9723, 1.0009)
	NNQE_v2 (CZ)	0.9866	0.9867	0.9878	0.9867	0.9867	0.9889 ± 0.00875	(0.9801, 0.9976)
	Quantum SVM	0.9721	0.9778	0.9836	0.9783	0.9775	0.9654 ± 0.0145	(0.951, 0.980)
	SVM (linear)	1.0000	0.9956	0.9960	0.9956	0.9956	0.9933 ± 0.005	(0.988, 0.998)
	SVM (poly)	0.9989	0.9911	0.9927	0.9911	0.9911	0.9944 ± 0.0045	(0.990, 0.999)
	SVM (rbf)	0.9654	0.9556	0.9712	0.9556	0.9539	0.9387 ± 0.0095	(0.929, 0.948)
	SVM (sigmoid)	0.0524	0.0133	0.9868	0.0133	0.0004	0.0524 ± 0.0035	(0.049, 0.056)
	KNN (k=3)	0.9967	0.9911	0.9927	0.9911	0.9911	0.9966 ± 0.0055	(0.991, 1.002)
	EfficientNetB0	0.992728	0.997039	0.994954	0.995084	0.994858	0.9970 ± 0.00305	(0.9940, 1.0001)
	VGG16	0.986793	0.99034	0.98796	0.984055	0.984605	0.9903 ± 0.00695	(0.9834, 0.9973)
**Apple Dataset**	NNQE_v1 (CNOT)	1.0000	0.9667	0.9694	0.9667	0.9664	0.9200 ± 0.0467	(0.8733, 0.9667)
	NNQE_v2 (CZ)	1.0000	0.9667	0.9697	0.9667	0.9665	0.9277 ± 0.0390	(0.8886, 0.9667)
	Quantum SVM	0.974576	0.933333	0.944444	0.92674	0.930723	0.9066 ± 0.0550	(0.852, 0.962)
	SVM (linear)	100.0000	94.5977	95.5586	93.9078	94.4328	94.598 ± 1.61	(92.99, 96.21)
	SVM (poly)	100.0000	97.2874	97.8632	96.9158	97.2174	97.287 ± 2.455	(94.83, 99.74)
	SVM (rbf)	96.7920	95.9770	96.0043	95.6044	95.5458	95.977 ± 4.805	(91.18, 100.78)
	SVM (sigmoid)	41.8915	41.8851	13.9617	33.3333	19.6764	41.885 ± 2.255	(40.63, 43.14)
	KNN (k=3)	0.972995	0.898851	0.909667	0.898851	0.895065	0.89885 ± 0.03265	(0.8672, 0.9305)
	EfficientNetB0	0.968461	0.953103	0.950398	0.953346	0.949464	0.9531 ± 0.0471	(0.9060, 1.0002)
	VGG16	0.960571	0.95977	0.955291	0.962442	0.956744	0.9598 ± 0.04525	(0.9145, 1.0050)

#### 4.3.1 Training dynamics and generalization stability.

Analysis of training and validation curves are illustrated in Fig 9, revealed divergent learning patterns between the hybrid models. NNQEv1 achieved rapid initial convergence but exhibited validation accuracy degradation in later epochs, signaling overfitting. Conversely, NNQEv2 demonstrated gradual yet stable convergence, maintaining consistent validation accuracy throughout training. This behavior implies enhanced regularization and generalization in the CZ-based architecture, potentially attributable to the gate’s inherent noise resilience in quantum simulations.

#### 4.3.2 MNIST dataset evaluation.

On the MNIST dataset, both hybrid quantum-classical models exhibited strong performance. The NNQEv1 model achieved a test accuracy of 98.58%, whereas NNQEv2 reached a slightly higher accuracy of 98.72% (Table 3). These results surpass the reported performance of VGG16 (97.35%) and EfficientNetB0 (98.1%), and are competitively close to the top-performing classical polynomial-kernel SVM (99.17%). Cross-validation analysis further highlights NNQEv2’s superior stability, with a mean accuracy of 98.16% ± 0.0005, indicating robust generalization. The purely quantum SVM was not evaluated on MNIST due to the high dimensionality of the feature space, which exceeds the feasible input size for current QSVM implementations.

#### 4.3.3 FruitQ dataset evaluation.

For the FruitQ dataset, both NNQEv1 and NNQEv2 attained identical test accuracies of 98.67%. Although slightly below the fine-tuned EfficientNetB0 (99.70%) and VGG16 (99.03%) models, the hybrid architectures substantially outperformed the purely quantum SVM and traditional baselines, such as SVM with an RBF kernel. Cross-validation metrics favored NNQEv2, which achieved a higher mean accuracy of 98.89% with a narrower confidence interval compared to NNQEv1 (98.66%), underscoring its enhanced generalization stability.

#### 4.3.4 Apple dataset evaluation.

The Apple dataset, characterized by limited sample size and class imbalance, provided a rigorous test of model robustness. Both hybrid models achieved a test accuracy of 96.67%, exceeding the performance of the purely quantum SVM (93.33%) and most classical SVM variants. While fine-tuned VGG16 (95.98%) and EfficientNetB0 (95.31%) demonstrated strong performance, the hybrid models remained highly competitive. Cross-validation analysis further distinguished NNQEv2, which attained a mean accuracy of 92.77% ± 0.0390, compared to NNQEv1’s 92.00% ± 0.0467, highlighting the hybrid architectures’ resilience and stability in data-scarce scenarios where large-scale pre-training is limited or infeasible.

#### 4.3.5 Cross-dataset insights: Accuracy differentials, variance, and confidence intervals

An examination of the comprehensive performance results, summarized in Table 3, reveals a structured hierarchy of model efficacy across the evaluated datasets. On data-rich benchmarks such as MNIST and FruitQ, classical architectures, notably EfficientNetB0 and Support Vector Machines (SVMs) with polynomial kernels, achieve the highest classification accuracies. This observation aligns with expectations, as these models effectively exploit large volumes of data to learn intricate feature representations.

Despite the high peak performance of classical models, the hybrid quantum-classical architectures exhibit notable competitiveness. On the MNIST dataset, NNQEv1 achieves a test accuracy of 98.58% while NNQEv2 reaches 98.72%, surpassing the reported performance of VGG16 (97.35%) and EfficientNetB0 (98.1%). For the FruitQ dataset, both NNQEv1 and NNQEv2 attain 98.67% test accuracy, approaching the performance of VGG16 (99.03%). These findings demonstrate that the proposed hybrid frameworks are viable alternatives to established deep learning models.

A critical observation arises from evaluating generalization stability, as quantified by the variance in 5-fold cross-validation accuracy. Across all three datasets, NNQEv2 consistently exhibits superior stability. For MNIST, NNQEv2’s mean CV accuracy of 98.16% ± 0.0005 shows a variance three times smaller than NNQEv1 (98.13% ± 0.0015) and substantially smaller than the SVM (poly) model (98.82% ± 0.005). Similarly, on the data-scarce Apple dataset, NNQEv2 (92.77% ± 0.0390) maintains a narrower confidence interval than NNQEv1 (92.00% ± 0.0467) and many classical models, highlighting its robustness under limited data conditions.

This empirically observed reduction in variance for the CZ-based NNQEv2 architecture supports the study’s central hypothesis. The CNOT gate in NNQEv1, by contrast, appears to introduce higher variability due to error propagation, whereas the CZ gate imparts an implicit regularization effect, enhancing generalization. While NNQEv2 does not universally surpass the highest-performing classical models in raw accuracy, it demonstrates highly consistent and reliable performance, indicating resilience to data variability—a desirable characteristic for specialized domains with limited training data.

It is important to note that all quantum machine learning (QML) models in this study were simulated on classical hardware. As quantum hardware continues to advance, real quantum implementation will become feasible, potentially further improving both accuracy and scalability.

Table 4 combines test accuracies between Adam, SGD, RMSprop, and Adamax optimizers for both models. Adam consistently achieves great performance, with accuracies of 96.67 % across settings. In contrast, SGD exhibits fluctuation, with accuracy dipping to 80.00 % in some cases. RMSprop is generally effective but less consistent, while Adamax performs similarly to Adam with high accuracies of 96.67%. NNQEv2 layers consistently outperform NNQEv1, notably with SGD, and match or exceed NNQEv1’s performance with Adam and Adamax. Overall, NNQEv2 performs similarly or even significantly better than NNQEv1, particularly in SGD settings, implying that NNQEv2 provides more consistent results across many optimizers.

**Table 4:** Test accuracy (%) of NNQEv1 and NNQEv2 with different optimizers across 1 to 5 layers on the apple dataset.

Layer	Optimizer			
	Adam	SGD	RMSprop	Adamax
**L1_NNQEv1**	96.67%	80.00%	96.67%	96.67%
**L2_NNQEv1**	93.33%	86.67%	93.33%	93.33%
**L3_NNQEv1**	96.67%	96.67%	96.67%	96.67%
**L4_NNQEv1**	96.67%	86.67%	96.67%	96.67%
**L5_NNQEv1**	96.67%	90.00%	96.67%	96.67%
**L1_NNQEv2**	96.67%	93.33%	96.67%	96.67%
**L2_NNQEv2**	96.67%	90.00%	96.67%	96.67%
**L3_NNQEv2**	96.67%	90.00%	89.99%	96.67%
**L4_NNQEv2**	96.67%	83.33%	93.33%	96.67%
**L5_NNQEv2**	96.67%	93.33%	93.33%	96.67%

## 5 Conclusions

This study conducted a systematic investigation into the impact of entangling gate selection within hybrid quantum-classical neural networks, specifically for the application of fruit quality assessment. The research, evaluated through quantum circuit simulations on classical hardware, compared two architectures: NNQEv1 (CNOT-based) and NNQEv2 (CZ-based). These models were benchmarked against a comprehensive suite of classical, quantum, and state-of-the-art deep learning models across three relevant datasets.

The central contribution of this work is the establishment of a rigorous theoretical basis for the superiority of the CZ gate for this task. The empirical results from the noiseless quantum simulations validated this theory, revealing a clear performance hierarchy. While state-of-the-art classical models like EfficientNetB0 delivered the highest peak accuracies on data-rich sets, the proposed hybrid models demonstrated significant advantages over traditional baselines like SVM and KNN. Most importantly, the CZ-based NNQEv2 model proved highly competitive with SOTA models on the data-scarce Apple dataset and consistently exhibited the lowest cross-validation variance across all scenarios. This superior generalization stability is the key distinguishing feature of the theoretically-grounded NNQEv2 architecture, confirming its reliability for fruit quality classification.

While the findings are based on quantum simulations performed on classical hardware, they highlight Quantum Machine Learning as a promising paradigm for complex classification tasks in agritech. The enhanced stability of the NNQEv2 architecture underscores the critical importance of hardware-aware, low-level design choices. As physical quantum hardware matures, the real implementation of models designed with such principles will become increasingly feasible, potentially offering robust and reliable solutions for automated fruit quality assessment. This work provides a foundational step in that direction, demonstrating that a theoretically-grounded gate choice can lead to measurably more stable models.

### 5.1 Future work and direction

Future efforts will focus on two primary directions. The first is to validate the robustness of the proposed architectures by moving beyond the current noiseless simulations. This will involve conducting comprehensive **noisy simulations** with hardware-calibrated error models, followed by the eventual deployment and evaluation on physical quantum processors. Incorporating noise-aware training protocols will be a key part of this effort.

The second direction is to extend the framework’s capabilities for broader agritech applications. This includes enabling the processing of full RGB image datasets for richer feature extraction and exploring more complex quantum circuits, such as those with larger convolution kernels (e.g., 3×3) and dynamic entanglement schemes for improved scalability. By pursuing these steps, the foundational insights from this work can be built upon to develop practical and robust quantum machine learning solutions for fruit quality assessment.

By pursuing these directions, the foundational insights from this work can be built upon to develop truly practical and robust quantum machine learning solutions for fruit quality assessment and other challenging classification tasks.
